# Supplementary material for: The asparagus genome sheds light on the origin and evolution of a young Y chromosome
Source: Nat Commun. 2017 Nov 2;8:1279. doi: 10.1038/s41467-017-01064-8 (PMC5665984; doi:10.1038/s41467-017-01064-8)
Supplement: Supplementary file 1 — Supplementary Information [file 41467_2017_1064_MOESM1_ESM.pdf]

Supplementary Note 1: DNA sequencing and assembly

Illumina sequencing and SOAPdenovo2 assembly

Nearly 341Gb of a variety of Illumina reads were generated for a single doubled haploid YY garden asparagus individual DH00/086, utilizing insert sizes that ranged from short insert paired-end libraries to 40kb mate-pair libraries (Supplementary Table 1).

Supplementary Table 1: Illumina sequence library statistics

Insert Size	Reads Length(bp)	Total Data(Gb)	Sequence Depth (X)	Physical Depth (X)
170bp	100	20.70	15.64	13.30
200bp	100	31.98	24.17	24.17
500bp	100	32.98	24.93	62.31
800bp	100	31.57	23.86	95.45
2Kb	90/49	51.44	38.88	563.48
5Kb	90	35.25	26.64	740.04
10Kb	90	32.41	24.49	1360.82
20Kb	90/49	83.73	63.28	7990.75
40Kb	90	21.83	16.50	3666.72
Total:	---	341.86	258.40	14517.04

Data were filtered before assembly using the following cutoffs:

(1): Reads with unidentified nucleotides (N) were removed. The 170nt library was filtered for reads >5% Ns, the 200bp library filtered for >10% Ns, and the 500 and 800nt libraries filtered for >2% Ns. The 2kb-40kb libraries were also filtered for >2% Ns. Filtration was adjusted based on the N nucleotide percentage of each library.

(2): Reads from short-insert libraries having more than 30%-40% bases with quality score less than 7, and reads from long-insert libraries that contained more than 60% bases with quality score less than 7.

(3): Reads with more than 10 bp aligned to the adapter sequence, allowing ≤ 2 bp mismatches.

(4): Small paired-end reads in short-insert libraries (except for paired-end reads from 170 bp insert library) that overlapped more than 10 bp with the corresponding paired end.

(5): Read1 and read2 of two paired-end reads that were completely identical (considered to be products of PCR duplication).

Supplementary Table 2: Filtered Illumina Read Statistics for DH00/086

Insert size (bp)	Read Length (bp)	Total Data (Gb)	Depth (X coverage)[1]
170	100	13.9	10.51
200	100	10.88	8.23
500	100	26.16	19.77
800	100	25.5	19.27
2,000	90/49	37.73	28.52
5,000	90	12.96	9.8
10,000	90	9.72	7.34
20,000	90/49	21.13	15.97
40,000	90	5.18	3.91
**Total:**		**163.15**	**123.32**

[1]Depth of coverage was calculated assuming a 1.32Gb genome size

After the above quality control and filtering steps, 163.15 Gb clean data was remained (Supplementary Table 2). For the clean data from short-insert size libraries (170bp, 200bp, 500bp, 800bp), a BGI custom program was used for reads trim and base correction. Then all the remained data was used for *de novo* genome assembly.

Briefly, we *de novo* assembled the garden asparagus genome using SOAPdenovo[34], and then used GapCloser (http://sourceforge.net/projects/soapdenovo2/files/GapCloser/) to fill gaps in scaffolds. To further scaffold and improve the contiguity of the assembly, we then used the SSPACE[35] scaffolder.

In more detail, SOAPdenovo operates by:

a. **Contig construction**: We first used all the reads from short-insert size libraries to construct a *de Bruijn* graph with *k*-mer parameter –K69 –R, then simplified the graphs by removing the tips and connections with low coverage, merging bubbles and masking small repeats, followed by connected the *k*-mer path to get a contig file.

b. **Scaffold construction:** All the usable reads were realigned onto the contig sequences, and the amount of shared paired-end relationships between each pair of contigs, as well as the rate of consistent and conflicting paired-ends, were calculated to construct the scaffolds in a stepwise fashion from short-insert size paired-ends to long-insert paired-ends.

c. **Gap filling**: To close the gaps inside the constructed scaffolds, which were mainly composed of repeats that were masked before scaffold construction, a BGI custom program KGF was first used to fill gaps based on short-insert size paired-ends reads. GapCloser was used to fill the gaps again based on the read pairs that had one end mapped to the unique contig and the others located in the gap region.

d. **Link scaffolds:** SSPACE was also used to improve the assembly. It used the overlap relationship of reads in contigs to extend the contigs, and the PE relationships to link the scaffolds again.

Finally, the initial Illumina-based assembly consisted of 112,602 contigs (≥ 100 bp) and 23,998 scaffolds (≥ 100 bp), with a contig N50 of 21.12 kb and scaffold N50 of 301.04kb (Supplementary Table 3).

All Illumina data used for genome assembly have been deposited in BioProject PRJNA317340.

Supplementary Table 3: The assembly statistics of the WGS results.

Metric	*Scaffold*		*Contig*	
	Size(bp)	Number	Size(bp)	Number
**N90**	73,440	4,140	5,762	53,430
**N80**	128,075	2,966	9,634	38,795
**N70**	178,416	2,199	13,335	29,055
**N60**	235,886	1,632	17,100	21,727
**N50**	301,040	1,196	21,179	15,892
**Longest**	1,874,998	--	176,610	--
**Total Size**	1,162,656,628	--	1,109,535,501	--
**Total Number(>=100bp)**	23,998	--	112,602	--
**Total Number(>=2Kb)**	9,610	--	75,256	--

PacBio sequencing and scaffold improvement

To improve the contiguity of the Illumina-based SOAPdenovo assembly, we generated Pacific Biosystems (PacBio) long-reads for further gap-filling and scaffolding[36]. High molecular weight DNA isolated from the reference DH00/086 individual was used as input for library preparation. The prepared library was size selected for >20kb fragments using the BluePippin (Sage Science, Beverly, MA) and sequenced on a PacBio RS II at the University of Florida Interdisciplinary Center for Biotechnology Research (ICBR). Nearly 6.07Gb of data were generated, and given that the garden asparagus genome is roughly 1.32Gb in size, the PacBio data corresponds to 4.60X coverage of the genome.

PBJelly2 (https://sourceforge.net/projects/pb-jelly/) version PBSuite_14.7.14 was used to improve the existing scaffold assembly with parameters "-minMatch 8 -sdpTupleSize 8 -minPctIdentity 75 -bestn 5 -nCandidates 10 -maxScore -500 –noSplitSubreads". Based on the PBJelly gap filling result, in order to fill the new introduced gaps and refine the assembly, we again used a BGI custom software KGF to fill gaps, and then used GapCloser to fill the gaps again with the short-insert size reads which had one end mapped to a unique contig and the other located in the gap regions.

For the PBJelly-improved Illumina assembly, the total assembled scaffold size is 1.18 Gb and contig size is 1.16 Gb. The contig N50, scaffold N50 of assembly are 88.16Kb and 303.52 Kb, respectively (scaffolds cut into contigs when Ns >=25bp) (Supplementary Table 4).

Supplementary Table 4: The assembly statistics after using Pacbio reads.
(Scaffold cuts into contigs when N >=25bp)

	Scaffold (bp)	Contig (bp)
Sequence number	23,968	42,527
Min	300	73
1st Qu.	473	691
Median	974	4,722
Mean	49,364	27,182
3rd Qu.	21,605	35,077
Max	1,881,081	503,700
Total	1,183,109,573	1,155,983,449
N50	303,519	88,165
N90	73,768	22,357
N95	37,344	12,180
Length>=2kb	9,804	25,057
GC percentage	0.38	0.39

Genetic mapping

To anchor the assembly onto pseudomolecules representing the 10 haploid chromosomes in garden asparagus, we utilized a doubled haploid population of 77 individuals to generate a genetic map by low depth resequencing. The doubled haploid individuals, all either XX or YY, were all derived from anther culture in a single XY male that was also sequenced. Additionally, the doubled haploid male and female parents of that XY male were also resequenced, so that all possible segregating alleles could be identified.

The 77 doubled haploid offspring were resequenced to an average of 3.5X coverage using PE150 reads on an Illumina NextSeq500 at the Georgia Genomics Facility at the University of Georgia. Briefly, libraries were sheared to an average of 700nt using a Covaris S-2, and 250ng of total DNA were used as input for the KAPA DNA library kit. All reads were aligned to the contigs using BWA version 0.7.10-r789 with default parameters. Samtools version 1.2 mpileup was used to combine the results for all individuals, retaining the read depth for each allele at each locus (-t DP) and filtered for polymorphic SNPs using bcftools version 1.2. SNPs with average read depth of <1 across the population, indels, SNPs with >2 alleles and SNPs with fewer than 5 non-reference sequence reads across the whole population were removed using vcftools v0.1.12a. Genotypes for each individual were called as AA, AB, BB or missing data using a script to process raw reads from the mpileup output, as the genotype calling function of bcftools sometimes imputes the incorrect genotype when read depth is low or absent.

Preliminary SNPs were further filtered by a script to remove all SNPs where the genotype quality score was <900. SNPs with >4 heterozygous individual scores called out of 77 were discarded. Hetrozygous SNPs should not exist in DH lines but sequencing errors and read mapping errors occur, and a strict limit of zero heterozygous SNPs removed too much otherwise good data. Furthermore SNPs with combined read depth <120 or >500 were removed (average 1.66x and 6.95x coverage). Low read depth tended to occur on the ends of contigs in the reference assembly and produced SNPs with too much missing data, while SNPs with >2x the average read depth tended to be due to multi-copy sequences and most could not be placed on the genetic map. The result of this filtering produced 3,352,321 individual SNPs that passed the quality checks, or an average of one SNP per 343bp.

SNPs from the same sequence contig were combined to create a consensus genotype for each contig, using the rule that a consensus genotype was assigned if >90% of the individual SNPs not including missing data from a contig had the same genotype. A genetic map was assembled using the consensus contig genotypes in an iterative process. A preliminary map was first generated using contigs with >100 SNPs and progressively refined using contigs with fewer SNPs. The genetic map was constructed and curated within Microsoft Excel as described previously[38]. The resulting genetic map of 649 distinct recombination patterns assembled into the expected 10 linkage groups. The genetic map contained 691 recombination events with no gap larger than 3 recombination events. After ordering the sequence contigs with the genetic map, any sequence scaffold could also be oriented relative to the genetic map if the scaffold spanned a recombination event for one or more individual in the mapping population.

The genetic mapping data were used to identify chimeric contigs or scaffolds in the genome assembly resulting from mis-assembly. Contigs with 5 or more consecutive SNPs that mapped to 2 different genetic loci >5cM apart were cut into separate contigs. Scaffolds that contained contigs mapping to different loci were broken. A total of 2503 chimeric joins between contigs within assembled sequence scaffolds were identified and broken to conform with the mapping data. An additional 361 chimeric joins were identified within assembled contigs. These were also broken and segments were placed in the correct recombination intervals.

As some of the regions of interest, notably the sex region, could have corresponded to large indels between the maternal and paternal haplotypes (i.e. hemizygous), scaffolds from these regions would not contain SNPs, and could not be placed on a SNP-based gentic map. Therefore, reads were mapped to the reference genome and sequence depth of coverage was calculated for each contig across the individuals of the mapping population (Supplementary Data 1). The sequence depth of coverage was then treated as a QTL used to aid mapping of 9,336 contigs that were identified with segments segregating for depth of coverage in the mapping population. These contigs totaled 259 MBP or 22.5% of the non-N sequence assembly. This is not to imply that 22.5% of the genome is hemizygous in the male or female parents. Parts of larger contigs could contain indels large enough to be detected by this approach, or the female sequence for a region was too diverged in for sequence reads to be mapped to the male reference genome. Of the contigs with putatively hemizygous segments, just 2367 were only mapped as depth-of-coverage QTLs and contained no mapped SNPs totaling 12Mbp or 1% of the sequence assembly. These include some of the scaffolds that were found to be perfectly co-segregating with sex.

BioNano optical mapping and super-scaffolding

To further improve the contiguity of the genome assembly, we generated BioNano optical maps for the reference YY individual as well as a sibling doubled haploid XX female (DH0094). High molecular weight DNA was isolated from frozen tissue and labeled with Nt.BspQI. For the YY male reference, nearly 88Gb (79X coverage) of single molecules greater than 150kb were generated. *De novo* assembly in IrysView software yielded a 1.205Gb optical assembly comprising 1,364 optical maps with an N50 size of 1.24Mb.

The scaffolds from the sequence genome assembly were mapped onto the optical map contigs using the Comparison tool in Irysview software (BioNano Genomics). The resulting super-scaffolds were then manually edited to remove sequence contigs where the genetic map data conflicted with the optical map assignments (see above). In cases where 2 or more sequence contigs mapped to the same part of an optical map, the sequence contig with the lower e-value was removed. In total 80.4% of the sequence assembly could be placed on the optical maps, and the remaining contigs were generally too small to contain enough restriction sites to assign a location. A second optical map of a female asparagus plant was used to order and orient a small fraction (<1%) of the genome that was genetically mapped but not oriented by the male optical map and genetic SNP map.

Combining genetic and optical map data, 93.7% of the assembled sequence was placed within recombination intervals on the 10 linkage groups in the genetic map. A total of 29.3% of assembled basepairs were ordered and oriented within recombination intervals. Ordered and oriented segments were put together as contiguous super-scaffolds within each recombination interval. Unordered scaffolds were placed within the genetic map at the distal end of each recombination interval. The majority of genetically mapped but unordered and unoriented pieces mapped to regions around the centromere that contained few recombination events, but about 1/3 of the genome assembly. Only 6.2% of the genome assembly could not be assigned to chromosomal locations in the linkage map. The assembled reference genome sequence was deposited in NCBI's genome database (accession number MPDI00000000.1) and the **Co**mparative **Ge**nomics Platform database (https://genomevolution.org/coge/)

Supplementary Figure 1: Pairwise LTR percent identities for 8,446 *Gypsy* and 10,891 *Copia* annotated retrotransposons. B) RepeatMasker annotation summary utilizing custom repeat database.

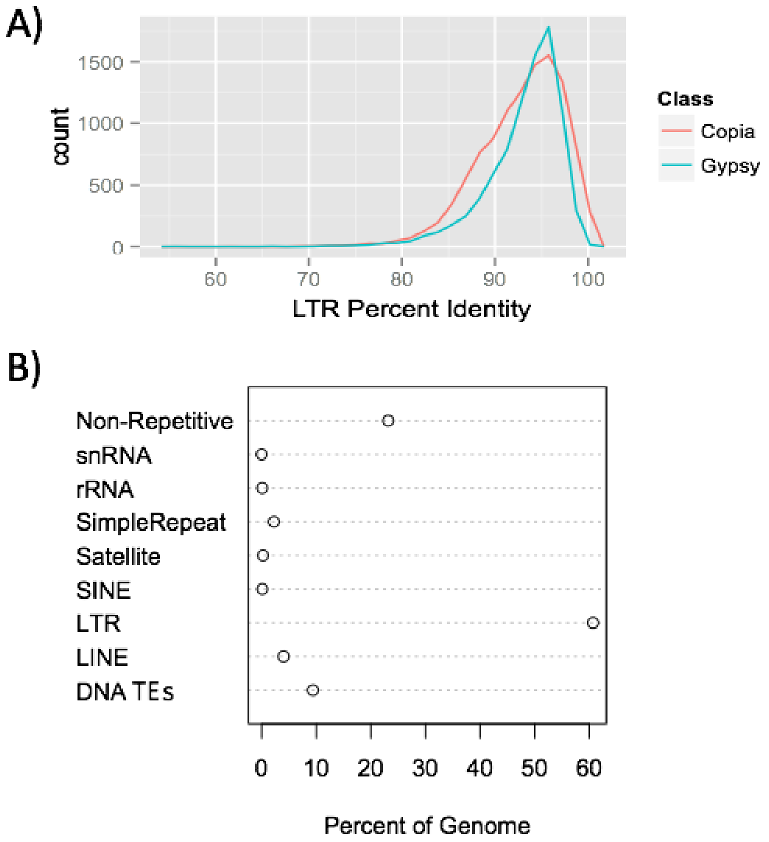

Supplementary Figure 2: Optical map alignment against garden asparagus YY reference pseudomolecules. Reference genome pseudomolecules are colored in green, while optical alignments are overlaid in blue. Darker blue shades represent overlaps of optical assemblies.

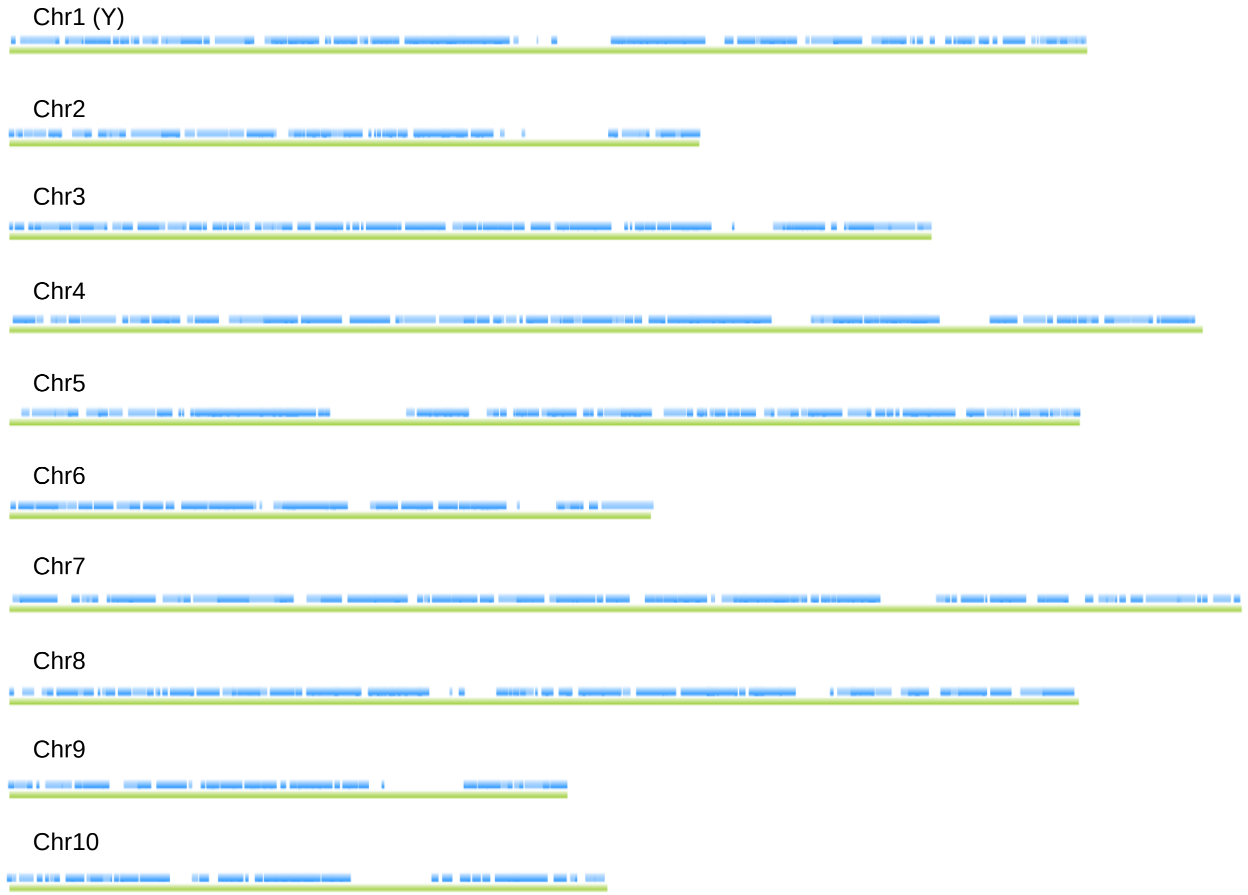

Supplementary Note 2 – Analysis of sex-linked region

Assembly of the non-recombining region of the Y

Leveraging the physical assembly, the genetic map, the optical maps, and Illumina resequencing of five female and four additional YY male garden asparagus accessions, we were able to contiguously assemble 847kb of largely hemizygous, non-recombining Y-specific sequence that is ordered and oriented in the genome assembly with hemizygosity boundaries between coordinates 3,411,433-4,258,465 of linkage group 1 (Figure 1B). Additional contigs mapping to this region were hemizygous but not ordered and oriented in the genetic map. Just as we did for all unordered contigs within all recombination intervals, these were placed at the distal end of the sex-linked interval from coordinates 4,330,815 to 5,457,683. Most of these contigs were small (0.15-65 kb, median = 10.8 kb) and did not align to the optical map scaffold that spanned the contiguous non-recombining region. Segments of these scaffolds did, however, exhibit hemizygosity or sex-specific SNPs in the comparison of 4 resequenced YY male and 5 resequenced XX female genomes. Focusing on annotated genes, an additional six gene models exhibit sex-specific alleles (no recombination between X and Y forms; AsparagusV1_01.247 - 01.248, and AsparagusV1_01.272-0.275) and twelve include polymorphisms that are shared between XX and YY accessions. Only one of these has an annotation (AsparagusV1_01.248; outer envelope protein 80, chloroplastic-like isoform X1). In summary we infer that the non-recombining sex determination region is less than 1 Mb is size (Figure 1B) including thirteen gene models that may be perfectly cosegregating with sex. These include the female suppressor (DUF 247 homolog), a male promoter (TDF1 homolog) and just four other gene models with BLASTX matches in the NCBI protein database.

Female suppressing gene identification

To identify a female suppressing gene on the Y chromosome, we identified and sequenced several independent hermaphrodite lines that were each derived from a different XY male:

1) Nearly 40,000 XY hybrid seeds were treated with cobalt-60 gamma irradiation to identify a single field-grown hermaphrodite mutant (G033) for whole genome resequencing. A wild type XY untreated control plant (K323) was also resequenced (Supplementary Figure 3).

2) Additional gamma irradiation produced 3 more mutants (Lim_mut1, Lim_mut3, and Lim_mut4), were male-to-female mutants. A control XY male plant (Lim_mut5) was also resequenced.

3) A natural XY male-to-hermaphrodite mutant (Line3) was discovered in a glasshouse. Floral bud RNA-seq of this individual, followed by genomic PCR-based confirmation, revealed a single base pair deletion in a coding exon of the SOFF female suppressor gene. This base pair deletion results in a frameshift mutation and a premature stop codon.

For each gamma irradiation individual, roughly 1ug of DNA was quantified using a Nanodrop ND-2000 and sheared to a mean fragment size of 650nt using a Covaris S-2. Sequencing libraries were prepared using the KAPA DNA Library Preparation Kit according to manufacturer instructions, performing 6 cycles of PCR to produce amplified library. Amplified libraries were sized on an Agilent DNA1000 chip, quantified using a Qubit, then pooled and sequenced on either an Illumina NextSeq500 for PE150 reads.

Raw reads were adapter clipped and quality trimmed using Trimmomatic v0.32, removing nucleotides from both the 5’ and 3’ ends with quality scores lower than Phred 10. A minimum length of 50nt was required. Cleaned reads were aligned to the reference pseudomolecules using bwa (v0.7.5a) with default options. Read trimming and alignment statistics are presented in Supplementary Table 5.

Supplementary Figure 3: Phenotypic identification of the gamma irradiated G033 male-to-hermaphrodite mutant. The resequencing of this genotype lead to identification of a single deletion interrupting a Y-specific gene encoding a DUF 247 containing protein.

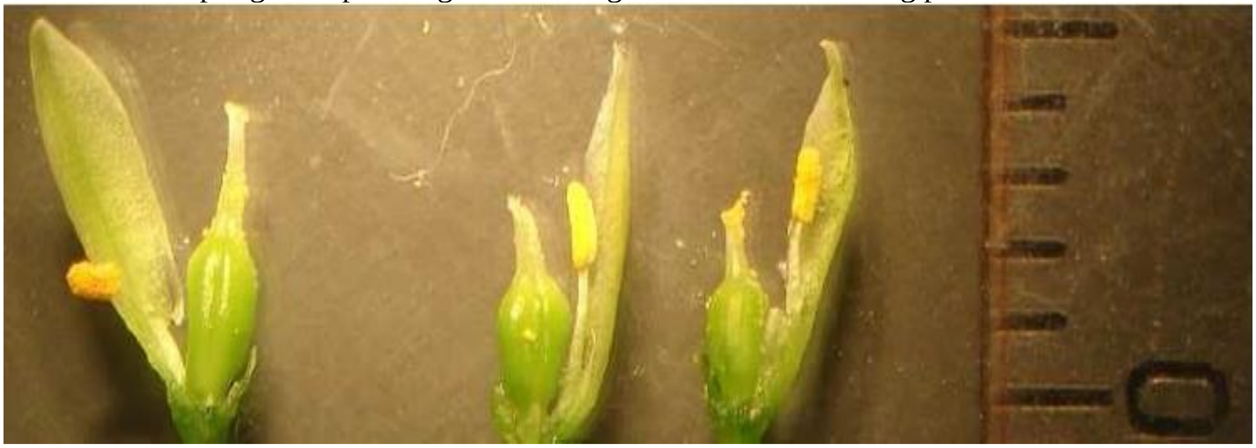

Supplementary Table 5: Sequencing and alignment results for irradiation mutants

Library	Mutation	Raw Pairs	Cleaned Pairs	Percent Aligned Concordantly
**Lim_mut1**	Male-to-Female	65,456,422	56,932,710	86.3%
**Lim_mut3**	Male-to-Female	64,855,322	56,532,689	87.7%
**Lim_mut4**	Male-to-Female	42,701,136	35,840,500	85.3%
**Lim_mut5**	XY Wildtype to Lim_mut1,2,3,4	72,789,286	59,670,777	84.3%
**G033**	Male-to-Herma	48,878,915	48,298,502	91.7%
**K323**	XY Wildtype to G033	45,874,881	45,421,305	92.1%

By manually viewing the alignments for the three male-to-female mutants (Lim_mut1, Lim_mut3, Lim_mut4), we identified that in each, the entire non-recombining region had been deleted on the Y, implicating the non-recombining region on the Y as being responsible for sex determination. After aligning the reads to the reference assembly, we used bedtools v2.25.0 intersect to calculate the read counts at every exon interval for G033 and K323, the first male-to-hermaphrodite mutant we identified. A single sex-linked exon had 0 read counts in G033, and >5 read counts in K323. This exon was part of a Y-linked gene with a PFAM annotation of “Domain of Unknown Function 247” (DUF247)

An additional spontaneous male-to-hermaphrodite mutant (Line3, Figure 2B; Supplementary Figure 4) was identified. Sanger verification of the SOFF gene in this individual shows a single base pair coding deletion relative to the reference genome sequence, highlighted in red below; this deletion leads to a frameshift mutation and premature stop codon in the SOFF gene.

Supplementary Figure 4: Alignment of the Line3 hermaphrodite SOFF CDS against the reference genome. Single base deletion is highlighted in red.

```
Line3           ATGTCTGAAGCCTGGGTTTCTCGATTGACATCGGATATAGGGTGGCTCAATAGCACAAAT
DH00/086        ATGTCTGAAGCCTGGGTTTCTCGATTGACATCGGATATAGGGTGGCTCAATAGCACAAAT
                ************************************************************

Line3           GCCCTGATGGCGGAGGCCTGGAGTCGTCATTCAATCTACGACGTACCAGACACATTCAAA
DH00/086        GCCCTGATGGCGGAGGCCTGGAGTCGTCATTCAATCTACGACGTACCAGACACATTCAAA
                ************************************************************

Line3           AGGATTAGCCCACAGATCCATAAGCCATCAACGTGCAGCATTGGACCACGGTACAATGGA
DH00/086        AGGATTAGCCCACAGATCCATAAGCCATCAACGTGCAGCATTGGACCACGGTACAATGGA
                ************************************************************

Line3           GATCTGAATCTCCTTCGTATGGAACGTCATAAACACAGGGCGCTACTGAACTTCCTCATC
DH00/086        GATCTGAATCTCCTTCGTATGGAACGTCATAAACACAGGGCGCTACTGAACTTCCTCATC
                ************************************************************

Line3           CGATGTCAAGTGTCGATCCATGACATCATACGAGCCCTGAGGAAGAACCTGCACGATTTC
DH00/086        CGATGTCAAGTGTCGATCCATGACATCATACGAGCCCTGAGGAAGAACCTGCACGATTTC
                ************************************************************

Line3           AGAGCCTGCTATCAAGATCTTGACACCTTTTGGATGAAGAATGATGATGAGTTCCTAAAA
DH00/086        AGAGCCTGCTATCAAGATCTTGACACCTTTTGGATGAAGAATGATGATGAGTTCCTAAAA
                ************************************************************

Line3           ATCATGATTTACGATGGGGCTTTCATGATTGAAATCATGATAGCGACCGTTGAACCATAT
DH00/086        ATCATGATTTACGATGGGGCTTTCATGATTGAAATCATGATAGCGACCGTTGAACCATAT
                ************************************************************

Line3           GAGCGCACACCTTCTAGCTATCATGCCAAGGACCCAATATTCAAGAAGCCATACTTGGTC
DH00/086        GAGCGCACACCTTCTAGCTATCATGCCAAGGACCCAATATTCAAGAAGCCATACTTGGTC
                ************************************************************

Line3           GAAGATCTTCGTGTAGATATGCTCAGGTTGGATAATCAAATTCCAA-GAAGGTCCTGGAG
DH00/086        GAAGATCTTCGTGTAGATATGCTCAGGTTGGATAATCAAATTCCAATGAAGGTCCTGGAG
                ********************************************** *************

Line3           ATATTGTCTAAATTCTGCAAGAACAAGGTAAGGAATGTTAATGAAATCTAAATCTTCATA
DH00/086        ATATTGTCTAAATTCTGCAAGAACAAGGTAAGGAATGTTAATGAAATCTAAATCTTCATA
                ************************************************************

Line3           CCTTGAAATGTCCCAGCTGTAACTCCAGAAGAACTTGCACAAAATTTTCATACCTCGTAA
DH00/086        CCTTGAAATGTCCCAGCTGTAACTCCAGAAGAACTTGCACAAAATTTTCATACCTCGTAA
                ************************************************************

Line3           TGCAAGATTAACCTAACAGTCAACGTTGTATGAAATGATACATTATGCAAGGAGGATACT
DH00/086        TGCAAGATTAACCTAACAGTCAACGTTGTATGAAATGATACATTATGCAAGGAGGATACT
                ************************************************************

Line3           CGCACAAAATTTGCTATCCATTGAAGATATCATTCCCATGAGATTTTTTTGCAAATAAAT
DH00/086        CGCACAAAATTTGCTATCCATTGAAGATATCATTCCCATGAGATTTTTTTGCAAATAAAT
                ************************************************************

Line3           GAGTAATGTGCATGCTCAAGAAAGCAGTCACCATATTGAAATCATGCAGAAGACACTATT
DH00/086        GAGTAATGTGCATGCTCAAGAAAGCAGTCACCATATTGAAATCATGCAGAAGACACTATT
                ************************************************************

Line3           TGCATATACATCATATATGCTCTATTGAACTAAGACCTGGATAAATAACTTCTGGATAGA
DH00/086        TGCATATACATCATATATGCTCTATTGAACTAAGACCTGGATAAATAACTTCTGGATAGA
                ************************************************************
```

```
Line3           TAAACTTTATTGAAATCCTGCAGAAGACACTATTTGCTGTTAAGTGTTGAACTTTGTGTA
DH00/086        TAAACTTTATTGAAATCCTGCAGAAGACACTATTTGCTGTTAAGTGTTGAACTTTGTGTA
                ************************************************************

Line3           TGGATCCCTTTATAAAGCTATCGTAATTTATGCTGTTGCTTAAAGCAAATAACTTTTTTT
DH00/086        TGGATCCCTTTATAAAGCTATCGTAATTTATGCTGTTGCTTAAAGCAAATAAC-TTTTTT
                ***************************************************** ******

Line3           CTGTCTTCCAGATCCAAAGCATTCATCAGCTGATCAGACATTTCTTCTTCCGCAAATATG
DH00/086        CTGTCTTCCAGATCCAAAGCATTCATCAGCTGATCAGACATTTCTTCTTCCGCAAATATG
                ************************************************************

Line3           AAGAGGGAAGATATGATATTAGCCAAACCTCTACGATATTTCACCTACCCGAGATAACAG
DH00/086        AAGAGGGAAGATATGATATTAGCCAAACCTCTACGATATTTCACCTACCCGAGATAACAG
                ************************************************************

Line3           GGCATCACCTACTGGATGTGTACAAAAAAACTCTTATACAGCATGGAGGTTATCATCACA
DH00/086        GGCATCACCTACTGGATGTGTACAAAAAAACTCTTATACAGCATGGAGGTTATCATCACA
                ************************************************************

Line3           CCAGCAGTCGCCAACCACTATCGGCAGTTGAACTACAGGAGGCGGGCGTAATTTTCCAGT
DH00/086        CCAGCAGTCGCCAACCACTATCGGCAGTTGAACTACAGGAGGCGGGCGTAATTTTCCAGT
                ************************************************************

Line3           GCAGTGAAACGCTGTCATTGACAGATATATGCTTCACCAAAGGTGTCCTTTGCCTACCTG
DH00/086        GCAGTGAAACGCTGTCATTGACAGATATATGCTTCACCAAAGGTGTCCTTTGCCTACCTG
                ************************************************************

Line3            CAGTCGACGTTGACGAAGCATTTGAAGTTGTTATGCGGAATCTCATTGCCTATGAGCAAG
DH00/086        CAGTCGACGTTGACGAAGCATTTGAAGTTGTTATGCGGAATCTCATTGCCTATGAGCAAG
                ************************************************************

Line3           CACATGGCGAAGGTCAAGAGGTAACATCCTATGTGTTTTTTATGGATGGCATTGTAAACA
DH00/086        CACATGGCGAAGGTCAAGAGGTAACATCCTATGTGTTTTTTATGGATGGCATTGTAAACA
                ************************************************************

Line3           ATGACAAAGATATTGCCTTGCTTCGAGAGAAGGGTATTATCAGGTCGGGGGTAAGCAGTG
DH00/086        ATGACAAAGATATTGCCTTGCTTCGAGAGAAGGGTATTATCAGGTCGGGGGTAAGCAGTG
                ************************************************************

Line3           ATAAGAGGATAGCCGATCTTTTTAATGGACTGACAAAAGGTATAGTTGCAAAAGTTGTCG
DH00/086        ATAAGAGGATAGCCGATCTTTTTAATGGACTGACAAAAGGTATAGTTGCAAAAGTTGTCG
                ************************************************************

Line3           ACAATGTTGATGTTGATGTAACCAAGGACATCAATGAGTATTGCAATAGAAGATGGAACA
DH00/086        ACAATGTTGATGTTGATGTAACCAAGGACATCAATGAGTATTGCAATAGAAGATGGAACA
                ************************************************************

Line3           GGTGGCAAGCCAACTTTAAGCAGAGATACTTTGCGAATCCATGGGTAACTTGCTCACTCA
DH00/086        GGTGGCAAGCCAACTTTAAGCAGAGATACTTTGCGAATCCATGGGTAACTTGCTCACTCA
                ************************************************************

Line3           TTGTAGGAGCTCTAGTATTAGGTCTCACCATCACTCAAACAATCTATGGCATCCTTTCTT
DH00/086        TTGTAGGAGCTCTAGTATTAGGTCTCACCATCACTCAAACAATCTATGGCATCCTTTCTT
                ************************************************************

Line3           ATAATAAGTGTAGTTAATGTAACTCTCATACTCGAAAATGTATGGATGATTCCAGTCTTG
DH00/086        ATAATAAGTGTAGTTAATGTAACTCTCATACTCGAAAATGTATGGATGATTCCAGTCTTG
                ************************************************************
```

```
Line3           ATCCCAGTCTTTTGTCATGGCTGTGTGGCTGTAAGCATTGTAATTTGAGACAATGACAAG
DH00/086        ATCCCAGTCTTTTGTCATGGCTGTGTGGCTGTAAGCATTGTAATTTGAGACAATGACAAG
                ************************************************************

Line3           GATGAATAGGCTAATATCAACTGAAAAAGCTTCATATTTTTTTTGGTTTTTGCTCAAGAT
DH00/086        GATGAATAGGCTAATATCAACTGAAAAAGCTTCATATTTTTTTTGGTTTTTGCTCAAGAT
                ************************************************************

Line3           TGAATAAAAATGGCTTTAAAAAGGTAATGTGTAACAATGTCTCAGATTTTATTCTTTCCA
DH00/086        TGAATAAAAATGGCTTTAAAAAGGTAATGTGTAACAATGTCTCAGATTTTATTCTTTCCA
                ************************************************************

Line3           CACCCTGAACTCAACTTGGTTGCTTCTGTGCCCAAGTCAAATATTAATAAAACCATGTGC
DH00/086        CACCCTGAACTCAACTTGGTTGCTTCTGTGCCCAAGTCAAATATTAATAAAACCATGTGC
                ************************************************************

Line3           CTTTCTTGCAAGATCTTGGATTTTGTTCGGGTGCCATTTAATAAGAGAAGCTTGAAGGCA
DH00/086        CTTTCTTGCAAGATCTTGGATTTTGTTCGGGTGCCATTTAATAAGAGAAGCTTGAAGGCA
                ************************************************************

Line3           TCCCTTTGCATTACCCAAATGGAATCTGGGAGCTATGTTTTAATTACATTTAAATAGTGG
DH00/086        TCCCTTTGCATTACCCAAATGGAATCTGGGAGCTATGTTTTAATTACATTTAAATAGTGG
                ************************************************************

Line3           CTCTTTGAGGGTGTTTGTCTTTCAAATGTTCAGTTCCATGTCTTACTTCCAGTTATAATT
DH00/086        CTCTTTGAGGGTGTTTGTCTTTCAAATGTTCAGTTCCATGTCTTACTTCCAGTTATAATT
                ************************************************************

Line3           TTTTCTAGTGTAGTGATACTCTTAGTTTCTGTTCATGATTTAATGCATAGTATCTGGCAA
DH00/086        TTTTCTAGTGTAGTGATACTCTTAGTTTCTGCTCATGATTTAATGCATAGTATCTGGCAA
                ******************************* ****************************

Line3           CCAATAAATGCAAATGCATTAGTGTCGATCACATAACAAGAAATGATTAGTTCTTCGAGC
DH00/086        CCAATAAATGCAAATGCATTAGTGTCGATCACATAACAAGAAATGATTAGTTCTTCGAGC
                ************************************************************

Line3           CAATTTAATTTTCTCTGTTATTCCTTGATTTCCAAAACTTTGTTATTGTTTTGTCTTATC
DH00/086        CAATTTAATTTTCTCTGTTATTCCTTGATTTCCAAAACTTTGTTATTGTTTTGTCTTATC
                ************************************************************

Line3           TGATCTCAGTCTTTCTTTGCAGGCCTTTCCCGGGATTCATAAATGTTGATCTCAACGGTA
DH00/086        TGATCTCAGTCTTTCTTTGCAGGCCTTTCCCGGGATTCATAAATGTTGATCTCAACGGTA
                ************************************************************

Line3           GGGTTTCGTGCTGGGGTTTGAGTATCTGTGGAGCATTTAGTGTGAGAAAACTGTGCTTAA
DH00/086        GGGTTTCGTGCTGGGGTTTGAGTATCTGTGGAGCATTTAGTGTGAGAAAACTGTGCTTAA
                ************************************************************

Line3           TTTCGCTTCTCCACTATGAGAGTGGAGGAGCACAACTAATGGTATCCAGTGTAAATTTAA
DH00/086        TTTCGCTTCTCCACTATGAGAGTGGAGGAGCACAACTAATGGTATCCAGTGTAAATTTAA
                ************************************************************

Line3           CTCTTTGTTTGTGGCTTGAGAACAACATGTTCTTTATATAGCCTTTGACAATGTAATAGA
DH00/086        CTCTTTGTTTGTGGCTTGAGAACAACATGTTCTTTATATAGCCTTTGACAATGTAATAGA
                ************************************************************

Line3           TAACATCAACTTCTTTGATACATACTAGCGATATTAGCATCCTATCTTTTTCTTCTTCAT
DH00/086        TAACATCAACTTCTTTGATACATACTAGCGATATTAGCATCCTATCTTTTTCTTCTTCAT
                ************************************************************

Line3           GTTAACAATCCTATAGCCTTTACACGGGTAATATATCCTTGTGATGTTTAATCCTAAGTT
DH00/086        GTTAACAATCCTATAGCCTTTACA------------------------------------
                ************************
```

```
Line3          TTTCTCTGGTCAGTGAATATGAACAGCAGCTATTATTTTATTGC
DH00/086       --------------------------------------------
```

Supplementary Note 3 – Accession resequencing and diversity analyses

DNA library preparation and analysis

To characterize the nucleotide variation across Y chromosomes from diverse germplasm in order to refine the recombination boundaries on the Y, we generated Illumina sequencing libraries for 4 XX/YY pairs spread across a SNP-based tree[69]. DNA was isolated from spear tissue using a Qiagen DNeasy Plant Mini kit. Roughly 1ug of DNA was quantified using a Nanodrop ND-2000 and sheared to a mean fragment size of 650nt using a Covaris S-2. Sequencing libraries were prepared using the KAPA DNA Library Preparation Kit according to manufacturer instructions, performing 6 cycles of PCR to produce amplified library. Amplified libraries were sized on an Agilent DNA1000 chip, quantified using a Qubit, then pooled and sequenced on either an Illumina NextSeq500 for SE150 reads.

Raw reads were adapter clipped and quality trimmed using Trimmomatic v0.32, removing nucleotides from both the 5' and 3' ends with quality scores lower than Phred 10. A minimum length of 50nt was required. Cleaned reads were aligned to the reference pseudomolecules using bwa (v0.7.5a) with default options (Supplementary Table 6).

Supplementary Table 6: Sequencing and Read Trimming Summary

Accession	Genotype	Read Length	Raw pairs	Clean pairs
**3594**	XX	SE150	59,539,933	59,120,181
**3315**	YY	SE150	59,638,369	59,638,231
**4808**	XX	SE150	44,087,819	44,084,145
**3559**	YY	SE150	69,768,794	69,752,287
**4746**	XX	SE150	73,518,788	73,471,742
**5491**	YY	SE150	69,026,512	69,024,162
**4790**	XX	SE150	39,653,256	39,651,799
**3214**	YY	SE150	80,718,517	80,701,537

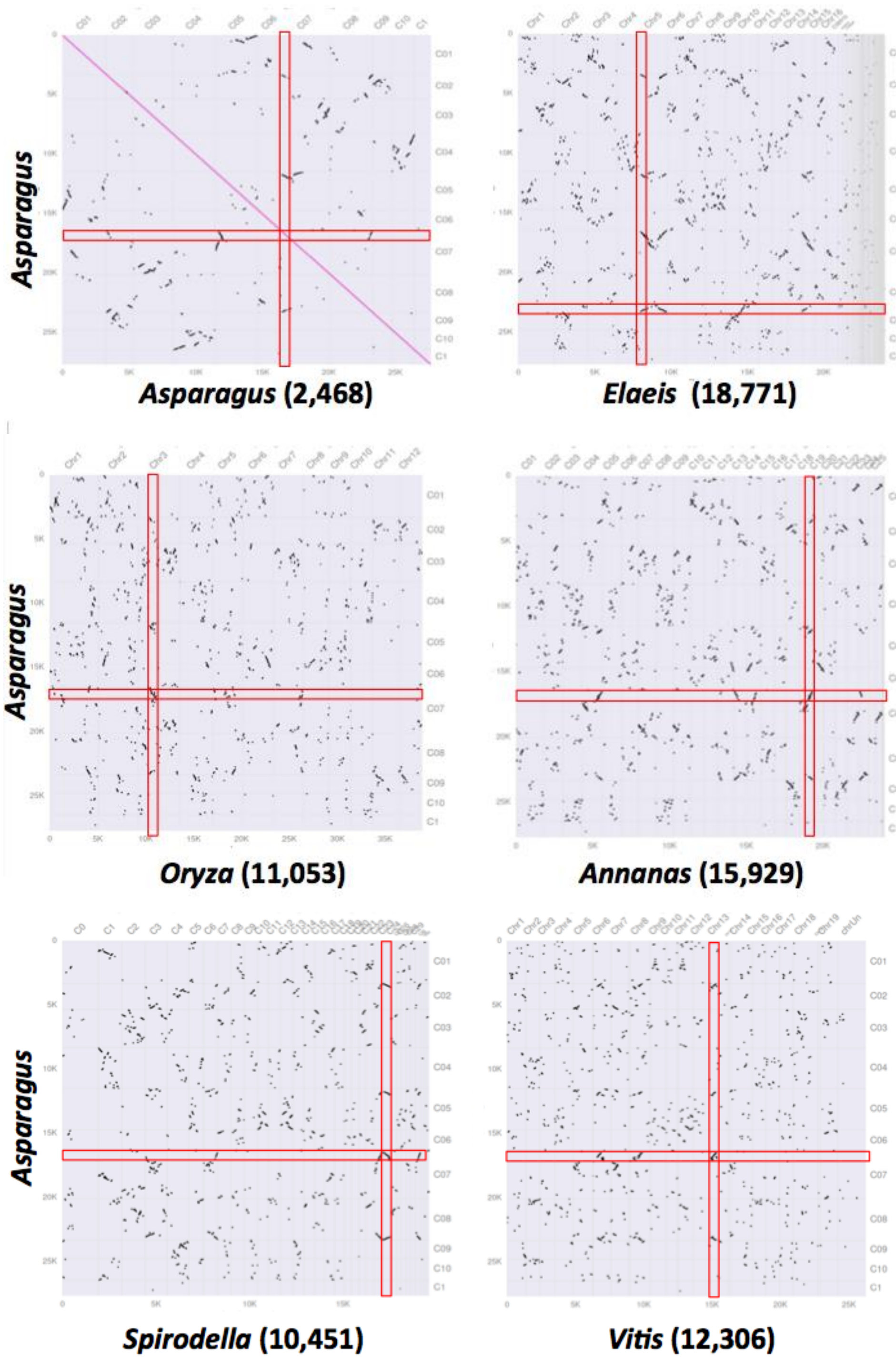

Supplementary Figure 5. Pairwise syntenic dot plots between garden asparagus and flowering plant genomes representing distinct lineages in the angiosperm phylogeny. Y-axes represent gene (left) and chromosome (right) number in the asparagus genome. *X*-axes represent gene (left) and chromosome or scaffold (right) numbers in the *Asparagus* (self comparison), *Elaeis*, *Oryza*, *Annanas*, *Spirodella* and *Vitis* genomes. Each dot represents a homologous gene pair retained in a synteny block with at least 4 gene pairs per block, and the total number of genes in syntenic blocks is shown in parentheses. Qualitative inspection of syntenic blocks suggests that syteny has persisted since two rounds of whole genome duplication (WGD) in the evolutionary history of the *Asparagus* lineage (see highligted in vertical red boxes). Phylogenomic analyses place these two events within the Asparagales since divergence of lineages leading to asparagus and the Orchidaceae. Horizontally highlighted boxes suggest two rounds of (WGD) in *Elaeis*, *Oryza*, *Annanas*, and Spirodella, and a genome triplication in *Vitis*, all supported by phylogenomic analyses (Figure 4).

Supplementary Table 7: Data sources for species used for gene family circumscription and analysis.

Species	Order	Data type	Data source	Reference
Amborella trichopoda	Amborellales	Genome	www.amborella.org	47
**Monocots**				
Acorus americanus	Acorales	RNA Seq assembly	asparagus.uga.edu/	This Study
Spirodella polyrhiza	Alismatales	Genome	phytozome.jgi.doe.gov	49
Zostera marina	Alismatales	Genome	www.ncbi.nlm.nih.gov/genome/2662	53
Dendrobium catenatum	Asparagales	Genome	www.ncbi.nlm.nih.gov/genome/31795	55
Phalaenopsis equestris	Asparagales	Genome	www.ncbi.nlm.nih.gov/genome/11403	42
Asparagus officinalis	Asparagales	Genome	asparagus.uga.edu/	This study
Asparagus asparagoides	Asparagales	RNA Seq assembly	asparagus.uga.edu/	16
Yucca aloifolia	Asparagales	RNA Seq assembly	asparagus.uga.edu/	This study
Elaeis guineensis	Arecales	Genome	genomsawit.mpob.gov.my/genomsawit/	50
Musa acuminata	Zingiberales	Genome	https://phytozome.jgi.doe.gov	39
Ananas comosus	Poales	Genome	www.ncbi.nlm.nih.gov/genome/genomes/13945	51
Oryza sativa	Poales	Genome	phytozome.jgi.doe.gov	41
Sorghum bicolor	Poales	Genome	phytozome.jgi.doe.gov	52
Brachypodium distachyon	Poales	Genome	phytozome.jgi.doe.gov	67
**Eudicots**				
Vitis vinifera	Vitales	Genome	phytozome.jgi.doe.gov	43
Arabidopsis thaliana	Brassicales	Genome	phytozome.jgi.doe.gov	40

Carica papaya	Brassicales	Genome	phytozome.jgi.doe.gov	68
Solanum lycopersicum	Solanales	Genome	phytozome.jgi.doe.gov	65

Supplementary Note 4 – Small RNA and PARE analysis

Small RNA-seq libraries and their pre-processing

Fifteen small RNA-sequencing libraries were prepared from vegetative and reproductive tissues including three libraries from leaf, shoot and root; four libraries from two distinct developmental stages (referred to as early and late) of male and female flowers; and eight libraries from male, female, and supermale spears of three different genotypes (Supplementary Table 8). These libraries were pre-processed using the script "prepro.py" version 0.2 (https://github.com/atulkakrana/helper.github) with default settings as described earlier[70-72]. Preprocessing included trimming of 3' adapters followed by filtering of reads shorter than 18 or longer than 36 nt for downstream analyses. Retained reads were then aligned to the asparagus genome using Bowtie (version - 0.12.8) with no mismatches allowed. Mapped reads from all sRNA libraries were then normalized to an empirically derived, 30 million reads base depth. A comparison of abundances by each small RNA size and by genomic features revealed no obvious differences between sexes or tissues (Supplementary Figure 6).

Supplementary Table 8: Small RNA sequencing statistics

Code	Title	Total Sequences	Genome Matched Reads	Distinct Genome Matched Reads
8A_Fs	Spears from a female plant, line 8A	51,071,610	44,292,038	12,135,137
8A_Ms	Spears from a male plant, line 8A	47,521,321	41,436,943	11,083,405
8A_SMs	Spears from a super male plant, line 8A	53,130,780	46,135,133	11,785,744
8B_Fs	Spears from a female plant, line 8B	49,631,096	42,729,231	9,414,643
8B_Ms	Spears from a male plant, line 8B	44,181,331	37,299,490	9,698,350
8B_SMs	Spears from a super male plant, line 8B	48,687,896	40,711,766	9,617,438
10_Fs	Spears from a female plant, line 10	53,644,329	44,772,773	10,880,427
10_Ms	Spears from a male plant, line 10	51,686,019	43,018,683	9,449,224
St_Aspa	Asparagus shoot	48,684,914	39,327,937	8,403,892
Lf_Aspa	Asparagus leaf	44,967,647	37,794,563	6,531,317
Rt_Aspa	Asparagus root	28,265,822	15,507,019	2,836,851
MFE_Aspa	Asparagus male flower early stage	40,926,331	33,222,626	8,900,510
MFM_Aspa	Asparagus male flower mid stage	56,362,666	46,016,681	10,164,868
FeFE_Aspa	Asparagus female flower early stage	43,941,398	35,539,006	9,231,377
FeFM_Aspa	Asparagus female flower mid stage	58,262,210	48,093,598	9,459,576

Supplementary Figure 6: The small RNA (sRNA) size and abundance distribution in distinct tissues of asparagus. Different colors represent genomic features to which sRNAs align: orange (intergenic), yellow (miRNAs), green (exons), brown (introns), light brown (repetitive elements), and unmapped (green). The X axis represents the size of the small RNA tag, while the Y axis is the normalized abundance of sRNA abundances in "Transcripts per 30 Million Mapped Reads" (TPM30).

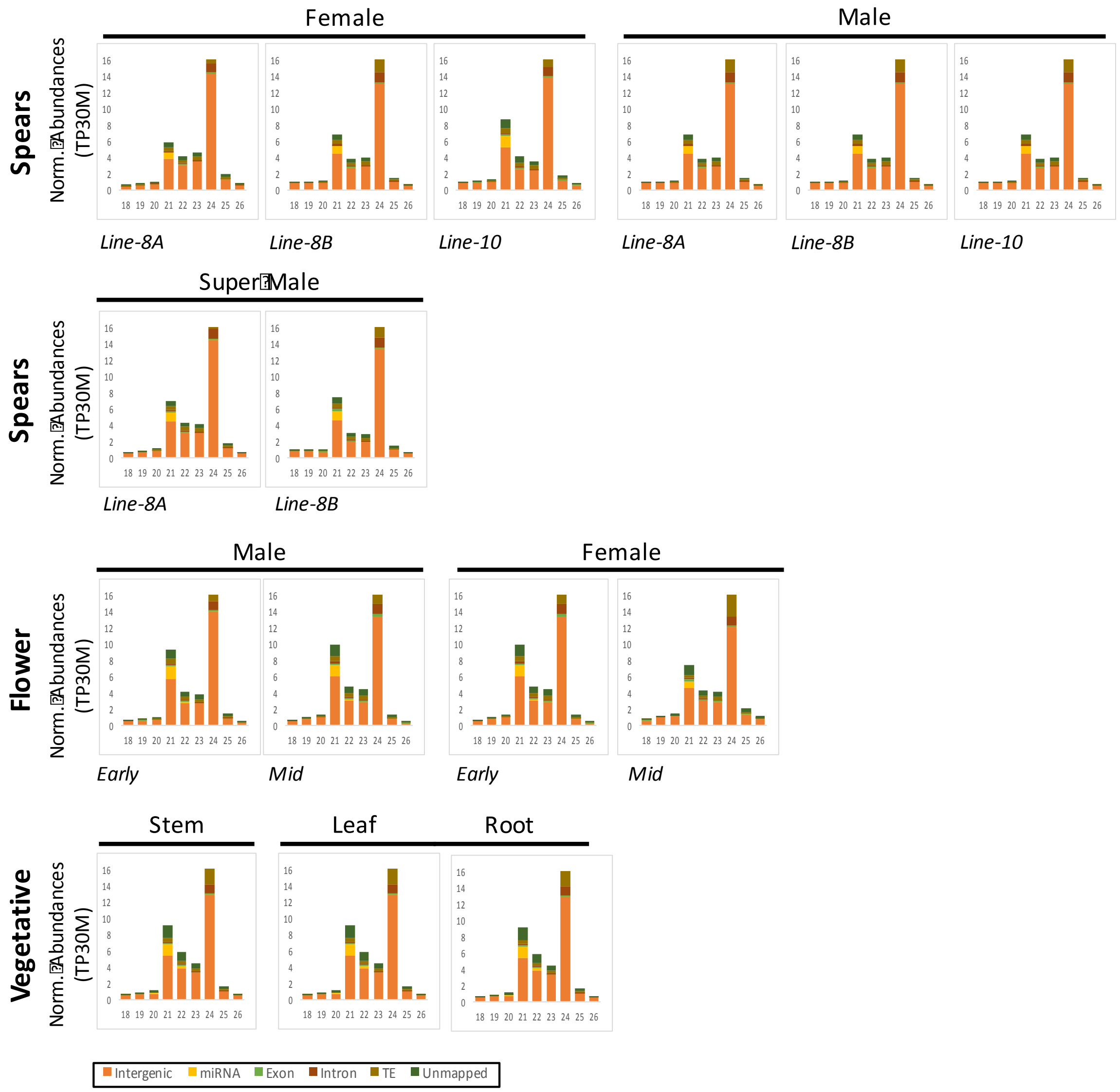

Small RNA distribution across sex-linked regions in asparagus

We assessed if there are any significant differences in production of sRNAs from the sex-linked regions of asparagus compared to the rest of genome. Since the abundance of many sRNAs may vary greatly between different tissues, we compared the sRNA distribution for sex-linked versus non-sex-linked regions separately, and for both reproductive and vegetative tissues. For this comparison, the following steps were performed:

1. The genome was computationally divided into two categories – sex-linked and other (non-sex-linked) regions.
2. The sequences in these two categories were further divided into three sub-categories based on mutually-exclusive, genome-wide features – exons, introns, and intergenic regions. We added one additional sub-category related to transposable elements that shared regions with exons/introns/intergenic regions
3. For each subcategory, we computed the hit-normalized abundances and counts of small RNAs of each size between 20 and 24-nt; we further normalized these to the total proportion of the genome (nucleotides) in every sub-category.
4. These values representing sRNA abundances and counts for different genomic features, from both categories – sex-linked and other regions – were used to compute Pearson's correlation and corresponding *p-values* using the *psych* R package (https://cran.r-project.org/web/packages/psych/index.html).

A high correlation ($\rho > 0.82$, $p < 0.05$) was observed for sRNAs (20-24 nucleotides) between feature pairs from the sex-linked region and the rest of the genome (Supplementary Figure 7).

Supplementary Figure 7: Heat map representing Pearson's correlation values for all-versus-all comparisons of the smRNA abundance levels from various genomic features of sex-liked (S.*) and non sex-linked (N.*) regions. The values highlighted with the black ovals represent the comparison between the same features of the sex-linked and non sex-linked regions ($p < 0.05$).

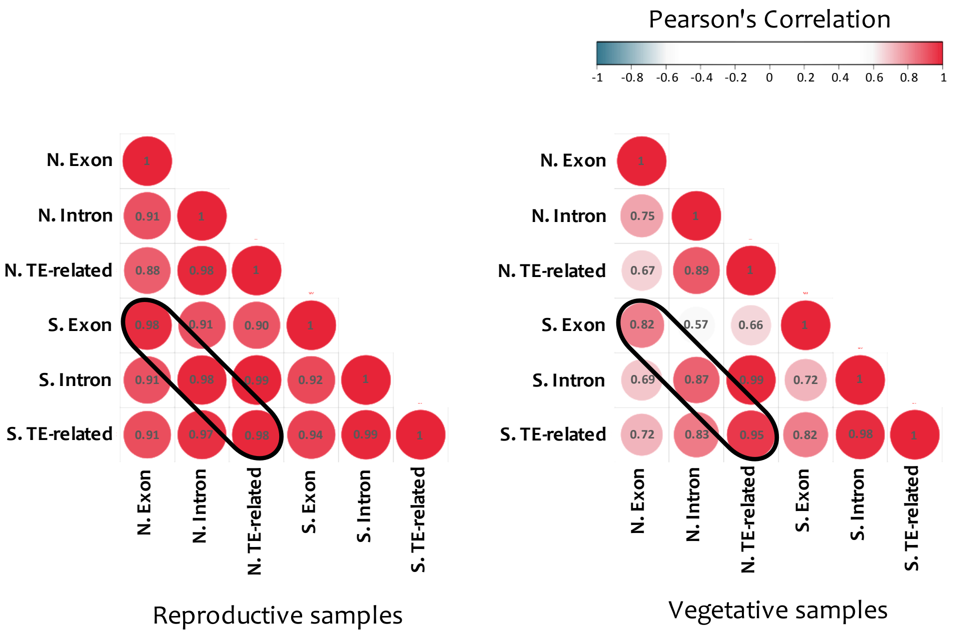

De novo identification of miRNA genes and their annotation

Mapped sRNA reads from all libraries were used as input to two different computational pipelines for discovery of miRNAs: i) a stringent pipeline for *de novo* identification, and ii) a relaxed pipeline for identification of conserved 'known' miRNAs. Steps in both pipelines involved processing using *perl* scripts as described earlier, with modified version of miREAP (https://sourceforge.net/projects/mireap/) and CentroidFold[73].

Using the 'stringent' criteria pipeline, sRNAs of length between 20 and 24 nt, with abundance >= 50 TP30M in at least one library, and total genome hits <= 20 were assessed for potential pairing of miRNA and miRNA* using miREAP and optimized for plant miRNA discovery with parameters *–d 400 –f 25*. Strand bias for precursors was computed as ratio of all reads mapped to sense strand against total reads mapped to both strands. In addition to strand bias, abundance bias was computed as ratio of two most abundant reads against all the reads mapped to same precursor. Candidate precursors with strand bias >= 0.9 and abundance bias >= 0.7 were selected, and foldback structure for precursor was predicted using CentroidFold. Each precursor was manually inspected to match criteria as described earlier[70]. All the miRNAs identified through this stringent pipeline were then annotated by matching mature sequences to miRBASE (version - 21), and those that did not matched to any known miRNA were considered as lineage or species-specific. In our 'relaxed' criteria pipeline, which is implemented to maximize identification of 'known' miRNAs; relaxed filters were applied – sRNAs between 20 and 24nt, with total genome hits <= 20 and abundance >= 15 TP30M, precursors with strand bias >= 0.7 and abundance bias >= 0.4. Stem-loop structures of candidate precursors were visually inspected, just as in the 'stringent' pipeline. Mature sequences of identified miRNAs were further matched with miRBASE entries (version-21), and those with total 'variance' (mismatches and overhangs) <= 4 were considered conserved miRNAs.

Both the 'stringent' and 'relaxed' criteria pipeline identified a total of 167 miRNA genes (including variants) with 105 non-redundant mature sequences corresponding to 78 distinct families (Supplementary Data 2). Out of these 78 families, 31 were already annotated in miRBASE (i.e. conserved families), and 47 families were lineage specific with a single miRNA member. For clarity, hereafter we will consider the non-redundant set of mature miRNAs (n=105) as total miRNAs identified in asparagus.

Out of 105 total miRNAs, four were significantly enriched (t-test, p <= 0.05) in flowers versus vegetative tissues – aof-miR8082, aof-miR2118a, aof-miR2275a, aof-miR394a; and nine were enriched (t-test, p <= 0.05) in spears from one of more genotype - aof-miR166c, aof-miR2118a, aof-miR2118b, aof-miR2118c, aof-miR2275c, aof-miR2275d, aof-mir8036, aof-mir8061 and aof-miR8080.

Sexual dimorphism in asparagus, along with the availability of male and female libraries generated in this study, enabled us to identify sex-specific expression patterns of miRNAs in spears from genotype-8A, 8B and 10 and flowers. A total 29 miRNAs displayed sex-biased expression in at least one genotype (Figure 3b) with 19 miRNAs enriched in male samples (FC>=4) while 16 depleted (FC >= -4) in male versus female spears from at least one asparagus line. In flower libraries (n=4), 9 miRNAs were found to be enriched while 6 depleted (absolute

FC >= 4) in at-least one developmental stage of male flower compared to female flower (Supplementary Table 9**)**. Interestingly, one copy of miR535 is generated from a Y-linked hemizygous region, showing 2- to 4-fold enrichment in two male spears; the locus is not single copy, however, and an autosomal copy on chromosome 9 maintains expression of miR535 in all samples tissues in both male and female. Evidence suggesting the possible roles of miRNAs in sex-specific flower development have increased in the past decade[74-76]; and a context-driven screening of miRNAs in a panel of dioecious and andromonoecious species can uncover additional underlying roles in future.

Supplementary Table 9: miRNA enrichment in floral vs vegetative tissues.

**Reproductive enriched**	aof-miR166c, aof-miR394a, aof-miR2118a, aof-miR2118b, aof-miR2118c, aof-miR2275a, aof-miR2275c, aof-miR2275d, aof-miR8036, aof-miR8061, aof-miR8082, aof-miR8080,
**Preferentially expressed in male and female flowers or spears**	aof-miR8002[1], aof-miR8005[3], aof-miR8007[1], aof-miR8025[3], aof-miR8026[1], aof-miR8044[1], aof-miR8047[1], aof-miR8049[1], aof-miR8061[1], aof-miR8066[1], aof-miR8073[3], aof-miR8076[3], aof-miR8078[1], aof-miR8080[1], aof-miR8085[3], aof-miR156a[3], aof-miR167b[1], aof-miR169c[1], aof-miR2118a[1], aof-miR2118b[1], aof-miR2118c[1], aof-miR2275a[1], aof-miR2275b[3], aof-miR2275d[3], aof-miR395a[1], aof-miR398a[3], aof-miR399a[1], aof-miR408a[1], aof-miR535a[1], aof-miR8019[2], aof-miR8051[2], aof-miR8052[2], aof-miR8068[2], aof-miR8070[2], aof-miR156b[2], aof-miR156c[2], aof-miR160a[2], aof-miR2275c[2], aof-miR4376a[2], aof-miR828a[2]
[1] differentially expressed in spears, [2]differentially expressed in flowers, [3]differentially expressed in both flower and spears	

Supplementary Figure 8: Presence and size of families that are identified in asparagus with other monocot and eudicot species with miRBASE entries. MicroRNA families from miRBASE that are present in at least three angiosperm species including asparagus are represented here. Darker color represents the abundance of miRNA family members in a given species.

	Eudicots									Monocots											Basal Angiosperm
	Asterids		Eurosids-I			Eurosids-II				Commelinids (grasses)								Non-Grass Sp.*			
Family	stu	sly	mdm	mtr	gma	ptc	ath	bra	ppt	hvu	tae	sbi	osa	bdi	zma	ata	sof	egu	asp	zmr	atr
miR156	18	7	31	16	28	12	15	14	3	2	1	9	19	17	23	10	1	0	10	5	4
miR159	0	1	3	2	10	5	4	1	0	2	2	2	7	9	22	0	5	0	2	1	1
miR160	3	1	5	6	7	11	5	2	9	0	1	6	12	11	13	6	0	0	5	3	1
miR164	2	4	6	4	11	6	5	9	0	0	1	5	6	7	16	6	0	0	4	3	2
miR166	7	4	9	9	26	17	10	0	13	3	0	11	24	18	26	10	0	0	10	4	4
miR167	8	3	10	3	11	11	6	4	1	0	3	9	16	10	20	12	2	0	6	2	1
miR168	0	4	2	4	2	4	4	6	0	2	0	1	3	2	4	2	2	0	2	1	1
miR169	14	6	6	15	25	36	18	0	0	1	1	18	22	20	32	19	0	0	9	2	3
miR171	9	5	15	9	27	17	6	5	2	2	2	11	14	8	28	8	0	0	4	3	3
miR172	10	2	15	6	15	12	8	7	0	0	0	6	5	4	8	6	0	6	5	1	1
miR319	5	4	3	8	17	9	3	2	7	0	1	2	4	3	8	2	0	0	3	0	5
miR390	2	4	6	1	9	5	4	2	4	0	0	1	2	2	4	2	0	0	1	2	2
miR393	2	0	6	3	12	5	4	0	0	0	0	2	3	3	6	2	0	0	3	2	1
miR394	0	2	2	0	9	4	3	0	0	0	0	2	1	1	4	2	0	0	1	0	1
miR395	10	2	9	15	13	11	6	8	1	0	2	12	25	28	32	12	0	0	7	0	1
miR396	2	3	7	5	15	9	4	2	0	0	1	5	13	10	13	10	1	0	6	2	5
miR398	4	0	3	4	4	4	6	2	0	0	1	1	2	2	4	4	0	0	1	0	1
miR399	29	1	10	22	9	10	7	0	0	1	1	11	11	4	20	4	0	0	3	2	0
miR408	4	0	4	2	7	2	2	2	2	0	1	1	2	2	3	2	5	0	1	0	0
miR482	9	7	5	2	9	8	0	0	0	0	0	0	0	0	2	0	0	0	2	0	0
miR535	0	0	4	0	0	0	0	0	4	0	0	0	2	0	0	0	0	0	2	0	1
miR827	2	0	1	0	0	1	1	0	0	0	0	0	1	2	2	0	0	0	1	0	0
miR828	0	0	2	0	2	2	1	0	0	0	0	0	0	0	0	0	0	0	1	0	1
miR2118	0	0	3	1	4	0	0	0	0	0	0	2	18	2	7	6	0	0	3	0	0
miR2275	0	0	0	0	0	0	0	0	0	0	1	0	4	3	8	6	0	0	4	0	0
miR4376	1	1	0	0	1	0	0	0	0	0	0	0	0	0	0	0	0	0	1	0	0

Species and their miRBASE codes used this figure: *Solanum tuberosum* (**stu**), *Solanum lycopersicum* (**sly**), *Malus domestica* (**mdm**), *Medicago truncatula* (**mtr**), *Glycine max* (**gma**), *Populus trichocarpa* (**ptc**), *Arabidopsis thaliana* (**ath**), *Brassica rapa* (**bra**), *Physcomitrella patens* (**ppt**), *Hordeum vulgare* (**hvu**), *Triticum aestivum* (**tae**), *Sorghum bicolor* (**sbi**), *Oryza sativa* (**osa**), *Brachypodium distachyon* (**bdi**), *Zea mays* (**zma**), *Aegilops tauschii* (**ata**), *Saccharum officinarum* (**sof**), *Elaeis guineensis* (**egu**), *Asparagus officinalis* (**asp**), *Zostera marina* (**zmr**), *Amborella trichopoda* (**atr**)

Targets of asparagus miRNAs

Targets for asparagus miRNAs were identified using the non-redundant miRNA set against the asparagus gene annotations and validated using Parallel Analysis of Read Ends (PARE) libraries. PARE sequencing profiles uncapped mRNA, products of cleavage or decay and facilitates validation of miRNA targets at genome or transcriptome level[77]. PARE libraries were prepared for the same samples from which sRNAs libraries were generated, which includes - flowers (male and female), leaf, root, shoot and spears (from male and female plants for three genotypes). Supplementary Table 10 includes the number of sequenced-, mapped-, and distinct-reads. These PARE libraries were pre-processed using the script "prepro.py" version 0.2 (https://github.com/atulkakrana/helper.github) with default settings which included adapter trimming, chopping of reads to 22nt length from 3' end and converting them to a condensed tab-separated file with distinct reads and abundances. These library specific tab-separated files were then used as an input to *sPARTA*[72], followed by target prediction and PARE-based cleavage validation using default *sPARTA* parameters *-genomeFeature 1 –tarPred E -tarScore --tag2FASTA --map2DD –validate* as described here https://github.com/atulkakrana/sPARTA.github/tree/master/sparta. The 'combined' result file containing non-redundant miRNA-target interactions for all sample-specific PARE libraries was then used to filter interactions with p-value <= 0.05 and PARE read depth >= 5 in at least one PARE library. A total of 239 PARE validated targets for 48 known miRNAs and 8 lineage-specific miRNAs were identified in this analysis. See Supplementary Data 3 for a complete list of targets and their annotations.

Supplementary Table 10: PARE library sequencing statistics

Code	Title	Total Sequences	Genome Matched Reads	Distinct Genome Matched Reads
8AF_d	Spears from a female plant, line 8A	22,588,072	18,009,472	5,120,005
8AM_d	Spears from a male plant, line 8A	25,441,003	20,220,850	5,756,795
8ASM_d	Spears from a super male plant, line 8A	20,141,905	16,066,842	4,435,997
8BF_d	Spears from a female plant, line 8B	21,201,482	16,656,489	4,993,526
8BM_d	Spears from a male plant, line 8B	19,713,775	15,518,414	5,178,914
8BSM_d	Spears from a super male plant, line 8B	22,629,307	18,002,585	5,022,696
10F_d	Spears from a female plant, line 10	26,367,163	20,496,145	6,143,532
10M_d	Spears from a male plant, line 10	21,334,611	16,555,039	5,335,536
AspM_mid	Male flower mid stage	28,944,491	23,471,432	7,667,051
AspM_ear	Male flower early stage	29,656,483	23,953,350	7,516,194
AspFM_mid	Female flower mid stage	25,144,959	20,389,279	6,741,041
AspFM_ear	Female flower early stage	23,728,164	19,220,692	6,678,085
Asp_shoot	Shoot	21,734,779	17,516,075	5,978,707
Asp_Lf	Leaf	24,820,749	20,086,939	6,155,838

The validated miRNA-target interactions were noticeably enriched for known miRNAs, with only 5% (n=13 out of 239) of these valid interactions corresponding to 16% (n=8 out of 49) of lineage specific miRNAs. Though some valid interactions of novel miRNAs have been missed due to the stringency of filters used, the difference in the number of validated miRNA-target interactions between known and novel miRNAs was significant. Overall, 226 interactions were validated for 48 conserved miRNAs belonging to 30 different families. This contrast in number of targets between novel and conserved miRNAs has been described earlier and indicates a general trend in land plants. The low number of validated targets for the novel miRNAs observed here and in earlier studies indicates possibility that either a portion of these miRNAs impart their functions by mechanism other than transcript cleavage or are yet to be integrated into the regulatory pathway.

Functional annotation of the validated targets was performed using BLAST2GO suite (version 3.0). Some targets that could not be annotated by BLAST2GO were assigned *ad hoc* annotations based on best *blastp* hit against Uniprotkb (E-value < 1e-5). As expected, targets of conserved miRNAs were mostly homologs of gene families that have been described in earlier studies (Supplementary Data 3; Supplementary Table 11)

Supplementary Table 11: PARE validated target families of conserved and novel miRNAs in Asparagus

Asparagus miRNA family	Conserved/Dominant Targets	Best BLASTp hit
**Targets for known/conserved miRNAs**		
**aof-miR156**	squamosa promoter-binding-like protein	
**aof-miR159**	transcription factor GAMYB-like protein	
**aof-miR160**	auxin response factor	
**aof-miR164**	NAC domain-containing protein	
**aof-miR166**	uridine kinase-like protein	
**aof-miR167**	auxin response factor	
**aof-miR168**	protein argonaute 1A/B-like proteins	
**aof-miR169**	phytochromobilin:ferredoxin oxidoreductase, chloroplastic	
**aof-miR171**	scarecrow-like protein	
**aof-miR172**	floral homeotic protein APETALA 2-like protein	
**aof-miR2118**	NAC transcription factor protein	
**aof-miR2275**	Multiple targets with no consensus	
**aof-miR319**	transcription factor TCP-like/transcription factor GAMYB-like/transcription factor PCF-like	
**aof-miR390**	leucine-rich repeat receptor protein kinase	
**aof-miR393**	transport inhibitor response like protein	
**aof-miR394**	F-box only protein	
**aof-miR395**	ATP-sulfurylase	
**aof-miR396**	growth-regulating factor protein	
**aof-miR398**	uridine 5'-monophosphate synthase	
**aof-miR408**	laccase-3-like protein	
**aof-miR477**	Multiple targets with no consensus	
**aof-miR482**	NAC transcription factor family	
**aof-miR535**	squamosa promoter-binding-like protein	
**aof-miR536**	Target not annotated, and no significant BLASTp results	
**aof-miR8155**	DDT domain-containing protein	
**aof-miR827**	Target not annotated	uncharacterized protein LOC103709368
**aof-miR828**	membrane-associated progesterone-binding protein	
**Targets for lineage-Specific Asparagus miRNAs**		
**aof-mir8038**	dna repair protein	
**aof-mir8062**	ubiquitin-like modifier-activating enzyme	
**aof-miR8003**	Target not annotated	ASIL1_ARATH Trihelix transcription factor ASIL1
**aof-miR8012**	Multiple targets with no consensus	
**aof-miR8068**	Target not annotated	Y5162_ARATH Uncharacterized protein At5g41620
**aof-miR8072**	Target not annotated	Y3720_ARATH UPF0481 protein At3g47200
**aof-miR8076**	probable serine threonine-protein kinase	
**aof-miR8082**	Target not annotated	UVR8_ARATH Ultraviolet-B receptor UVR8

Comparison of asparagus miRNA families with other Angiosperms

The asparagus includes 78 distinct miRNA families, out of which sixteen families are conserved across gymnosperms and angiosperms, and at least two are specific to monocots. Comparison of asparagus miRNA families to those present in at least three monocot species in miRBASE revealed thirteen monocot families that seem to be missing in asparagus (Supplementary Table 12). Assuming the possibility that these could have been missed due to parameters used in miRNA prediction (such as strand and abundance bias), we attempted to detect these families by reverse matching mature miRNA sequences from miRBASE to the sRNAs which are expressed about background levels (>= 15 TP30M) in at least one asparagus sRNA library. Candidate sRNAs that matched with <= 4 'variance' (mismatches and overhangs) to the mature miRNA entries for green plants in miRBASE were then mapped to the asparagus genome, followed by the manual inspection of their loci for stem-loop structure and the position of candidate sRNAs in predicted structure. Though we could find a few sRNAs matching the missing families in this analysis, none of these had acceptable stem-loop structure, supporting the absence of these miRNAs in the current asparagus genome assembly.

The monocot families missing in asparagus included miR397 and miR528. These miRNAs are present in *Z. marina*[49] and *M. acuminata*[39] representing distinct monocot orders (Alismatales and Zingiberales, repsectively), therefore suggesting the absence of these two miRNAs could just be restricted to asparagus and its close relatives. Of the remaining 11 miRNAs missing from asparagus, 8 are specific to the Poales (Supplementary Table 12**)**. The remaining three families, which include miR162, miR529 and miR530 are missing from *Z. marina* and *A. trichopoda* as well, suggesting they may be dispensable.

Besides the loss of miRNA families that are generally conserved across gymnosperms and angiosperms, asparagus also shows acquisition of eight miRNA families that have not been identified in the *Z. marina* nor *A. trichopoda*[47] genomes (Supplementary Table 12). These include two families - miR408 and miR827, both generated from single loci in asparagus, these are also present in *M. acuminata* and the *Poaceae* family, thus indicating they may have originated within the monocots after the lineage leading to the last common ancestors of the Asparagales and commelinids diverged from the lineage leading to *Z. marina*. Three other families, miR4376, miR5139 and miR8155 had only been reported in eudicots but haven now been identified in asparagus, suggesting that they originated in the last common ancestor of monocots and eudicots but lost in the other sequenced monocot lineages.

The miR482 and miR2118 families are largely conserved across gymnosperms and angiosperms[78]; their absence in *Z. marina* and *M. acuminata* suggest that these are either lost in certain monocot clades during monocot diversification or missed in genomic screening perhaps because these were not present above background level in tissues used to generate sRNA libraries. Unlike miR482 and miR2118, the miR2275 family has only been described in grasses until now. Improved phylogenetic sampling of additional monocot species is necessary to elucidate the gain, loss, diversification and function of these miRNAs.

Asparagus displays an impressive expansion of members and/or loci in at least fourteen miRNA families when compared with *Z. marina* and *A. trichopoda*. Expansion of miRNA families that

are ubiquitously expressed across land plants has been shown to occur via genome, segmental and tandem duplications[78,79]. Out of these expanded fourteen families, six show significant expansion (3 to 7 fold) (Supplementary Table 12).

Supplementary Table 12: MicroRNA families from monocots that either expanded or were acquired; and those that are missing in Asparagus

miRNA Family	Status in Asparagus	Presence in dicots	Presence in monocots after Asparagus speciation	Last common ancestor in
**miR156**	Expanded	Yes	Yes	Embryophytes[1]
**miR160**	Expanded	Yes	Yes	Embryophytes[1]
**miR162**	Missing	Yes	Yes	Gymnosperms[2]
**miR166**	Expanded	Yes	Yes	Embryophytes[1]
**miR167**	Expanded	Yes	Yes	Angiosperms[1]
**miR169**	Expanded	Yes	Yes	Angiosperms[1]
**miR172**	Expanded	Yes	Yes	Angiosperms[1]
**miR395**	Expanded	Yes	Yes	Angiosperms[1]
**miR397**	Lost	No	Yes	Angiosperms[1]
**miR408**	Gained	Yes	Yes	Embryophytes[1]
**miR437**	Missing	No	Yes	Poales
**miR444**	Missing	No	Yes	Poales
**miR482**	Gained	Yes	Yes	Gymnosperms[2]
**miR528**	Lost	No	Yes	Gymnosperms[2]
**miR529**	Missing	No	Yes	Embryophytes[2]
**miR530**	Missing	Yes	Yes	Angiosperms*
**miR531**	Missing	No	Yes	Poales
**miR827**	Gained	Yes	Yes	Gymnosperms[2]
**miR1122**	Missing	No	Yes	Poales
**miR1432**	Missing	No	Yes	Poales
**miR2118***	Gained	No	Yes	Gymnosperms[2]
**miR2275***	Gained	No	Yes	Monocots
**miR4376**	Gained	Yes	No	Eudicots
**miR5049**	Missing	No	Yes	Poales
**miR5062**	Missing	No	Yes	Poales
**miR6201**	Missing	No	Yes	Poales
**miR5139**	Gained	Yes	No	Eudicots
**miR8155**	Gained	Yes	No	Eudicots

[1]According to D'Hont et al. 2012[37]
[2]According to Chavez Montes et al. 2014[81]
*Observations based on unpublished data (A. Kakrana, J. Leebens-Mack and B. Meyers, in prep.)
Last common ancestors are based on sRNA libraries and miRBASE entries of available species
Expanded miRNAs increased in number of loci from 2 to 5 fold compared to Zostera and/or Amborella
Missing are those that are present in monocots, especially after speciation of Asparagus
Lost in Asparagus, evidences of there presence in other monocots, before or after the speciation of Asparagus exists
Gained in Asparagus, and were missing in older angiosperms - Amborella and Zostera

Supplementary References

67. The International *Brachypodium* Initiative, Genome sequencing and analysis of the model grass *Brachypodium distachyon*. *Nature*. **463**, 763–768 (2010).

68. R. Ming *et al.*, The draft genome of the transgenic tropical fruit tree papaya (*Carica papaya* Linnaeus). *Nature*. **452**, 991–996 (2008).

69. F. Mercati *et al.*, Single nucleotide polymorphism isolated from a novel EST dataset in garden asparagus (*Asparagus officinalis* L.). *Plant Sci.* **203-204**, 115–23 (2013).

70. Jeong, D.-H. *et al.* Massive analysis of rice small RNAs: mechanistic implications of regulated microRNAs and variants for differential target RNA cleavage. *Plant Cell* **23,** 4185–4207 (2011).

71. Patel, P., Ramachandruni, S. D., Kakrana, A., Nakano, M. & Meyers, B. C. miTRATA: a web-based tool for microRNA Truncation and Tailing Analysis. *Bioinforma. Oxf. Engl.* **32,** 450–452 (2016).

72. Kakrana, A., Hammond, R., Patel, P., Nakano, M. & Meyers, B. C. sPARTA: a parallelized pipeline for integrated analysis of plant miRNA and cleaved mRNA data sets, including new miRNA target-identification software. *Nucleic Acids Res.* **42,** e139–e139 (2014).

73. Sato, K., Hamada, M., Asai, K. & Mituyama, T. CENTROIDFOLD: a web server for RNA secondary structure prediction. *Nucleic Acids Res.* **37,** W277–280 (2009).

74. Aukerman, M. J. & Sakai, H. Regulation of flowering time and floral organ identity by a MicroRNA and its APETALA2-like target genes. *Plant Cell* **15,** 2730–2741 (2003).

75. Schmid, M. *et al.* Dissection of floral induction pathways using global expression analysis. *Dev. Camb. Engl.* **130,** 6001–6012 (2003).

76. Chuck, G., Meeley, R., Irish, E., Sakai, H. & Hake, S. The maize tasselseed4 microRNA controls sex determination and meristem cell fate by targeting Tasselseed6/indeterminate spikelet1. *Nat. Genet.* **39,** 1517–1521 (2007).

77. German, M. A., Luo, S., Schroth, G., Meyers, B. C. & Green, P. J. Construction of Parallel Analysis of RNA Ends (PARE) libraries for the study of cleaved miRNA targets and the RNA degradome. *Nat. Protoc.* **4,** 356–362 (2009).

78. Chávez Montes, R. A. *et al.* Sample sequencing of vascular plants demonstrates widespread conservation and divergence of microRNAs. *Nat. Commun.* **5,** 3722 (2014).

79. Maher, C., Stein, L. & Ware, D. Evolution of Arabidopsis microRNA families through duplication events. *Genome Res.* **16,** 510–519 (2006).
