# Supplementary material for: The asparagus genome sheds light on the origin and evolution of a young Y chromosome
Source: Nat Commun. 2017 Nov 2;8:1279. doi: 10.1038/s41467-017-01064-8 (PMC5665984; doi:10.1038/s41467-017-01064-8)
Supplement: Supplementary file 3 — Description of Additional Supplementary Files [file 41467_2017_1064_MOESM3_ESM.pdf]

Description of Additional Supplementary Files

File Name: Supplementary Data 1
Description: LinkageMapCoverage.xlsx.gz - A gzipped excel file with read coverage and genotyping data for the double haploid population used the construct the genetic map.

File Name: Supplementary Data 2
Description: miRNAs.xlsx - An excel file with all microRNA annotated in the Asparagus genome.

File Name: Supplementary Data 3
Description: miRNAtargets.xlsx - and excel file with microRNA targets identified with PAR reads.
